# Supplementary material for: HIVprotI: an integrated web based platform for prediction and design of HIV proteins inhibitors
Source: J Cheminform. 2018 Mar 9;10:12. doi: 10.1186/s13321-018-0266-y (PMC5845081; doi:10.1186/s13321-018-0266-y)
Supplement: Supplementary file 2 — Additional file 2. Source code of HIVProtI web server. [file 13321_2018_266_MOESM2_ESM.zip › HIVprotI_Source-code/hivprotI/weka-3-6-6/doc/weka/experiment/RandomSplitResultProducer.html]

RandomSplitResultProducer


---


|  |  |  |
| --- | --- | --- |
| **Overview** \| **Package** \| **Class** \| **Tree** \| **Deprecated** \| **Index** \| **Help** \| **Weka's home** | |  |
| **PREV CLASS**   **NEXT CLASS** | **FRAMES**    **NO FRAMES**     **All Classes** |  |
| SUMMARY: NESTED \| FIELD \| CONSTR \| METHOD | DETAIL: FIELD \| CONSTR \| METHOD |  |


---


## weka.experiment Class RandomSplitResultProducer

```
java.lang.Object
  weka.experiment.RandomSplitResultProducer
```

**All Implemented Interfaces:**: java.io.Serializable, AdditionalMeasureProducer, OptionHandler, RevisionHandler, ResultProducer

---

``` public class RandomSplitResultProducer extends java.lang.Object implements ResultProducer, OptionHandler, AdditionalMeasureProducer, RevisionHandler ```

Generates a single train/test split and calls the appropriate SplitEvaluator to generate some results.


Valid options are:

```
 -P <percent>
  The percentage of instances to use for training.
  (default 66)
```

```
 -D
 Save raw split evaluator output.
```

```
 -O <file/directory name/path>
  The filename where raw output will be stored.
  If a directory name is specified then then individual
  outputs will be gzipped, otherwise all output will be
  zipped to the named file. Use in conjuction with -D. (default splitEvalutorOut.zip)
```

```
 -W <class name>
  The full class name of a SplitEvaluator.
  eg: weka.experiment.ClassifierSplitEvaluator
```

```
 -R
  Set when data is not to be randomized and the data sets' size.
  Is not to be determined via probabilistic rounding.
```

```
 Options specific to split evaluator weka.experiment.ClassifierSplitEvaluator:
```

```
 -W <class name>
  The full class name of the classifier.
  eg: weka.classifiers.bayes.NaiveBayes
```

```
 -C <index>
  The index of the class for which IR statistics
  are to be output. (default 1)
```

```
 -I <index>
  The index of an attribute to output in the
  results. This attribute should identify an
  instance in order to know which instances are
  in the test set of a cross validation. if 0
  no output (default 0).
```

```
 -P
  Add target and prediction columns to the result
  for each fold.
```

```
 Options specific to classifier weka.classifiers.rules.ZeroR:
```

```
 -D
  If set, classifier is run in debug mode and
  may output additional info to the console
```

All options after -- will be passed to the split evaluator.

**Version:**
:   $Revision: 6255 $

**Author:**
:   Len Trigg (trigg@cs.waikato.ac.nz)

**See Also:**: Serialized Form

---

| **Field Summary** | |
| --- | --- |
| `static java.lang.String` | `DATASET_FIELD_NAME`             The name of the key field containing the dataset name |
| `static java.lang.String` | `RUN_FIELD_NAME`             The name of the key field containing the run number |
| `static java.lang.String` | `TIMESTAMP_FIELD_NAME`             The name of the result field containing the timestamp |


**Constructor Summary**

`RandomSplitResultProducer()`


| **Method Summary** | |
| --- | --- |
| `void` | `doRun(int run)`             Gets the results for a specified run number. |
| `void` | `doRunKeys(int run)`             Gets the keys for a specified run number. |
| `java.util.Enumeration` | `enumerateMeasures()`             Returns an enumeration of any additional measure names that might be in the SplitEvaluator |
| `java.lang.String` | `getCompatibilityState()`             Gets a description of the internal settings of the result producer, sufficient for distinguishing a ResultProducer instance from another with different settings (ignoring those settings set through this interface). |
| `java.lang.String[]` | `getKeyNames()`             Gets the names of each of the columns produced for a single run. |
| `java.lang.Object[]` | `getKeyTypes()`             Gets the data types of each of the columns produced for a single run. |
| `double` | `getMeasure(java.lang.String additionalMeasureName)`             Returns the value of the named measure |
| `java.lang.String[]` | `getOptions()`             Gets the current settings of the result producer. |
| `java.io.File` | `getOutputFile()`             Get the value of OutputFile. |
| `boolean` | `getRandomizeData()`             Get if dataset is to be randomized |
| `boolean` | `getRawOutput()`             Get if raw split evaluator output is to be saved |
| `java.lang.String[]` | `getResultNames()`             Gets the names of each of the columns produced for a single run. |
| `java.lang.Object[]` | `getResultTypes()`             Gets the data types of each of the columns produced for a single run. |
| `java.lang.String` | `getRevision()`             Returns the revision string. |
| `SplitEvaluator` | `getSplitEvaluator()`             Get the SplitEvaluator. |
| `static java.lang.Double` | `getTimestamp()`             Gets a Double representing the current date and time. |
| `double` | `getTrainPercent()`             Get the value of TrainPercent. |
| `java.lang.String` | `globalInfo()`             Returns a string describing this result producer |
| `java.util.Enumeration` | `listOptions()`             Returns an enumeration describing the available options.. |
| `java.lang.String` | `outputFileTipText()`             Returns the tip text for this property |
| `void` | `postProcess()`             Perform any postprocessing. |
| `void` | `preProcess()`             Prepare to generate results. |
| `java.lang.String` | `randomizeDataTipText()`             Returns the tip text for this property |
| `java.lang.String` | `rawOutputTipText()`             Returns the tip text for this property |
| `void` | `setAdditionalMeasures(java.lang.String[] additionalMeasures)`             Set a list of method names for additional measures to look for in SplitEvaluators. |
| `void` | `setInstances(Instances instances)`             Sets the dataset that results will be obtained for. |
| `void` | `setOptions(java.lang.String[] options)`             Parses a given list of options. |
| `void` | `setOutputFile(java.io.File newOutputFile)`             Set the value of OutputFile. |
| `void` | `setRandomizeData(boolean d)`             Set to true if dataset is to be randomized |
| `void` | `setRawOutput(boolean d)`             Set to true if raw split evaluator output is to be saved |
| `void` | `setResultListener(ResultListener listener)`             Sets the object to send results of each run to. |
| `void` | `setSplitEvaluator(SplitEvaluator newSplitEvaluator)`             Set the SplitEvaluator. |
| `void` | `setTrainPercent(double newTrainPercent)`             Set the value of TrainPercent. |
| `java.lang.String` | `splitEvaluatorTipText()`             Returns the tip text for this property |
| `java.lang.String` | `toString()`             Gets a text descrption of the result producer. |
| `java.lang.String` | `trainPercentTipText()`             Returns the tip text for this property |

**Methods inherited from class java.lang.Object**

`equals, getClass, hashCode, notify, notifyAll, wait, wait, wait`

**Field Detail**

### DATASET\_FIELD\_NAME

```
public static java.lang.String DATASET_FIELD_NAME
```

:   The name of the key field containing the dataset name

---


### RUN\_FIELD\_NAME

```
public static java.lang.String RUN_FIELD_NAME
```

:   The name of the key field containing the run number

---


### TIMESTAMP\_FIELD\_NAME

```
public static java.lang.String TIMESTAMP_FIELD_NAME
```

:   The name of the result field containing the timestamp


**Constructor Detail**

### RandomSplitResultProducer

```
public RandomSplitResultProducer()
```


**Method Detail**

### globalInfo

```
public java.lang.String globalInfo()
```

:   Returns a string describing this result producer

    :   **Returns:**: a description of the result producer suitable for displaying in the explorer/experimenter gui

---


### setInstances

```
public void setInstances(Instances instances)
```

:   Sets the dataset that results will be obtained for.

    :   **Specified by:**: `setInstances` in interface `ResultProducer`
    :   **Parameters:**: `instances` - a value of type 'Instances'.

---


### setAdditionalMeasures

```
public void setAdditionalMeasures(java.lang.String[] additionalMeasures)
```

:   Set a list of method names for additional measures to look for
    in SplitEvaluators. This could contain many measures (of which only a
    subset may be produceable by the current SplitEvaluator) if an experiment
    is the type that iterates over a set of properties.

    :   **Specified by:**: `setAdditionalMeasures` in interface `ResultProducer`
    :   **Parameters:**: `additionalMeasures` - an array of measure names, null if none

---


### enumerateMeasures

```
public java.util.Enumeration enumerateMeasures()
```

:   Returns an enumeration of any additional measure names that might be
    in the SplitEvaluator

    :   **Specified by:**: `enumerateMeasures` in interface `AdditionalMeasureProducer`
    :   **Returns:**: an enumeration of the measure names

---


### getMeasure

```
public double getMeasure(java.lang.String additionalMeasureName)
```

:   Returns the value of the named measure

    :   **Specified by:**: `getMeasure` in interface `AdditionalMeasureProducer`
    :   **Parameters:**: `additionalMeasureName` - the name of the measure to query for its value **Returns:**: the value of the named measure **Throws:**: `java.lang.IllegalArgumentException` - if the named measure is not supported

---


### setResultListener

```
public void setResultListener(ResultListener listener)
```

:   Sets the object to send results of each run to.

    :   **Specified by:**: `setResultListener` in interface `ResultProducer`
    :   **Parameters:**: `listener` - a value of type 'ResultListener'

---


### getTimestamp

```
public static java.lang.Double getTimestamp()
```

:   Gets a Double representing the current date and time.
    eg: 1:46pm on 20/5/1999 -> 19990520.1346

    :   **Returns:**: a value of type Double

---


### preProcess

```
public void preProcess()
                throws java.lang.Exception
```

:   Prepare to generate results.

    :   **Specified by:**: `preProcess` in interface `ResultProducer`
    :   **Throws:**: `java.lang.Exception` - if an error occurs during preprocessing.

---


### postProcess

```
public void postProcess()
                 throws java.lang.Exception
```

:   Perform any postprocessing. When this method is called, it indicates
    that no more requests to generate results for the current experiment
    will be sent.

    :   **Specified by:**: `postProcess` in interface `ResultProducer`
    :   **Throws:**: `java.lang.Exception` - if an error occurs

---


### doRunKeys

```
public void doRunKeys(int run)
               throws java.lang.Exception
```

:   Gets the keys for a specified run number. Different run
    numbers correspond to different randomizations of the data. Keys
    produced should be sent to the current ResultListener

    :   **Specified by:**: `doRunKeys` in interface `ResultProducer`
    :   **Parameters:**: `run` - the run number to get keys for. **Throws:**: `java.lang.Exception` - if a problem occurs while getting the keys

---


### doRun

```
public void doRun(int run)
           throws java.lang.Exception
```

:   Gets the results for a specified run number. Different run
    numbers correspond to different randomizations of the data. Results
    produced should be sent to the current ResultListener

    :   **Specified by:**: `doRun` in interface `ResultProducer`
    :   **Parameters:**: `run` - the run number to get results for. **Throws:**: `java.lang.Exception` - if a problem occurs while getting the results

---


### getKeyNames

```
public java.lang.String[] getKeyNames()
```

:   Gets the names of each of the columns produced for a single run.
    This method should really be static.

    :   **Specified by:**: `getKeyNames` in interface `ResultProducer`
    :   **Returns:**: an array containing the name of each column

---


### getKeyTypes

```
public java.lang.Object[] getKeyTypes()
```

:   Gets the data types of each of the columns produced for a single run.
    This method should really be static.

    :   **Specified by:**: `getKeyTypes` in interface `ResultProducer`
    :   **Returns:**: an array containing objects of the type of each column. The objects should be Strings, or Doubles.

---


### getResultNames

```
public java.lang.String[] getResultNames()
```

:   Gets the names of each of the columns produced for a single run.
    This method should really be static.

    :   **Specified by:**: `getResultNames` in interface `ResultProducer`
    :   **Returns:**: an array containing the name of each column

---


### getResultTypes

```
public java.lang.Object[] getResultTypes()
```

:   Gets the data types of each of the columns produced for a single run.
    This method should really be static.

    :   **Specified by:**: `getResultTypes` in interface `ResultProducer`
    :   **Returns:**: an array containing objects of the type of each column. The objects should be Strings, or Doubles.

---


### getCompatibilityState

```
public java.lang.String getCompatibilityState()
```

:   Gets a description of the internal settings of the result
    producer, sufficient for distinguishing a ResultProducer
    instance from another with different settings (ignoring
    those settings set through this interface). For example,
    a cross-validation ResultProducer may have a setting for the
    number of folds. For a given state, the results produced should
    be compatible. Typically if a ResultProducer is an OptionHandler,
    this string will represent the command line arguments required
    to set the ResultProducer to that state.

    :   **Specified by:**: `getCompatibilityState` in interface `ResultProducer`
    :   **Returns:**: the description of the ResultProducer state, or null if no state is defined

---


### outputFileTipText

```
public java.lang.String outputFileTipText()
```

:   Returns the tip text for this property

    :   **Returns:**: tip text for this property suitable for displaying in the explorer/experimenter gui

---


### getOutputFile

```
public java.io.File getOutputFile()
```

:   Get the value of OutputFile.

    :   **Returns:**: Value of OutputFile.

---


### setOutputFile

```
public void setOutputFile(java.io.File newOutputFile)
```

:   Set the value of OutputFile.

    :   **Parameters:**: `newOutputFile` - Value to assign to OutputFile.

---


### randomizeDataTipText

```
public java.lang.String randomizeDataTipText()
```

:   Returns the tip text for this property

    :   **Returns:**: tip text for this property suitable for displaying in the explorer/experimenter gui

---


### getRandomizeData

```
public boolean getRandomizeData()
```

:   Get if dataset is to be randomized

    :   **Returns:**: true if dataset is to be randomized

---


### setRandomizeData

```
public void setRandomizeData(boolean d)
```

:   Set to true if dataset is to be randomized

    :   **Parameters:**: `d` - true if dataset is to be randomized

---


### rawOutputTipText

```
public java.lang.String rawOutputTipText()
```

:   Returns the tip text for this property

    :   **Returns:**: tip text for this property suitable for displaying in the explorer/experimenter gui

---


### getRawOutput

```
public boolean getRawOutput()
```

:   Get if raw split evaluator output is to be saved

    :   **Returns:**: true if raw split evalutor output is to be saved

---


### setRawOutput

```
public void setRawOutput(boolean d)
```

:   Set to true if raw split evaluator output is to be saved

    :   **Parameters:**: `d` - true if output is to be saved

---


### trainPercentTipText

```
public java.lang.String trainPercentTipText()
```

:   Returns the tip text for this property

    :   **Returns:**: tip text for this property suitable for displaying in the explorer/experimenter gui

---


### getTrainPercent

```
public double getTrainPercent()
```

:   Get the value of TrainPercent.

    :   **Returns:**: Value of TrainPercent.

---


### setTrainPercent

```
public void setTrainPercent(double newTrainPercent)
```

:   Set the value of TrainPercent.

    :   **Parameters:**: `newTrainPercent` - Value to assign to TrainPercent.

---


### splitEvaluatorTipText

```
public java.lang.String splitEvaluatorTipText()
```

:   Returns the tip text for this property

    :   **Returns:**: tip text for this property suitable for displaying in the explorer/experimenter gui

---


### getSplitEvaluator

```
public SplitEvaluator getSplitEvaluator()
```

:   Get the SplitEvaluator.

    :   **Returns:**: the SplitEvaluator.

---


### setSplitEvaluator

```
public void setSplitEvaluator(SplitEvaluator newSplitEvaluator)
```

:   Set the SplitEvaluator.

    :   **Parameters:**: `newSplitEvaluator` - new SplitEvaluator to use.

---


### listOptions

```
public java.util.Enumeration listOptions()
```

:   Returns an enumeration describing the available options..

    :   **Specified by:**: `listOptions` in interface `OptionHandler`
    :   **Returns:**: an enumeration of all the available options.

---


### setOptions

```
public void setOptions(java.lang.String[] options)
                throws java.lang.Exception
```

:   Parses a given list of options.


    Valid options are:

    ```
     -P <percent>
      The percentage of instances to use for training.
      (default 66)
    ```

    ```
     -D
     Save raw split evaluator output.
    ```

    ```
     -O <file/directory name/path>
      The filename where raw output will be stored.
      If a directory name is specified then then individual
      outputs will be gzipped, otherwise all output will be
      zipped to the named file. Use in conjuction with -D. (default splitEvalutorOut.zip)
    ```

    ```
     -W <class name>
      The full class name of a SplitEvaluator.
      eg: weka.experiment.ClassifierSplitEvaluator
    ```

    ```
     -R
      Set when data is not to be randomized and the data sets' size.
      Is not to be determined via probabilistic rounding.
    ```

    ```
     Options specific to split evaluator weka.experiment.ClassifierSplitEvaluator:
    ```

    ```
     -W <class name>
      The full class name of the classifier.
      eg: weka.classifiers.bayes.NaiveBayes
    ```

    ```
     -C <index>
      The index of the class for which IR statistics
      are to be output. (default 1)
    ```

    ```
     -I <index>
      The index of an attribute to output in the
      results. This attribute should identify an
      instance in order to know which instances are
      in the test set of a cross validation. if 0
      no output (default 0).
    ```

    ```
     -P
      Add target and prediction columns to the result
      for each fold.
    ```

    ```
     Options specific to classifier weka.classifiers.rules.ZeroR:
    ```

    ```
     -D
      If set, classifier is run in debug mode and
      may output additional info to the console
    ```

    All options after -- will be passed to the split evaluator.

    :   **Specified by:**: `setOptions` in interface `OptionHandler`
    :   **Parameters:**: `options` - the list of options as an array of strings **Throws:**: `java.lang.Exception` - if an option is not supported

---


### getOptions

```
public java.lang.String[] getOptions()
```

:   Gets the current settings of the result producer.

    :   **Specified by:**: `getOptions` in interface `OptionHandler`
    :   **Returns:**: an array of strings suitable for passing to setOptions

---


### toString

```
public java.lang.String toString()
```

:   Gets a text descrption of the result producer.

    :   **Overrides:**: `toString` in class `java.lang.Object`
    :   **Returns:**: a text description of the result producer.

---


### getRevision

```
public java.lang.String getRevision()
```

:   Returns the revision string.

    :   **Specified by:**: `getRevision` in interface `RevisionHandler`
    :   **Returns:**: the revision


---


|  |  |  |
| --- | --- | --- |
| **Overview** \| **Package** \| **Class** \| **Tree** \| **Deprecated** \| **Index** \| **Help** \| **Weka's home** | |  |
| **PREV CLASS**   **NEXT CLASS** | **FRAMES**    **NO FRAMES**     **All Classes** |  |
| SUMMARY: NESTED \| FIELD \| CONSTR \| METHOD | DETAIL: FIELD \| CONSTR \| METHOD |  |


---
